# Thymol and carvacrol against *Klebsiella*: anti-bacterial, anti-biofilm, and synergistic activities—a systematic review

**DOI:** 10.3389/fphar.2024.1487083

**Published:** 2024-10-24

**Authors:** Kousha Farhadi, Erta Rajabi, Hesam Aldin Varpaei, Maryam Iranzadasl, Sepideh Khodaparast, Mohammadreza Salehi

**Affiliations:** ^1^ Faculty of Medicine, Tehran University of Medical Sciences (TUMS), Tehran, Iran; ^2^ College of Nursing, Michigan State University, East Lansing, MI, United States; ^3^ Department of Traditional Medicine, School of Persian Medicine, Shahed University, Tehran, Iran; ^4^ Neuroscience Institute, Tehran University of Medical Sciences (TUMS), Tehran, Iran; ^5^ Research Center for Antibiotic Stewardship and Antimicrobial Resistance, Department of Infectious Diseases, Imam Khomeini Hospital Complex, Tehran University of Medical Sciences (TUMS), Tehran, Iran

**Keywords:** *Klebsiella*, *K. pneumoniae*, antimicrobial resistance, thymol, carvacrol, synergistic, biofilm

## Abstract

**Introduction:**

*Klebsiella* poses a significant global threat due to its high antibiotic resistance rate. In recent years, researchers have been seeking alternative antimicrobial agents, leading to the introduction of natural compounds such as monoterpenes, specifically thymol and carvacrol. This review aims to illustrate the potential antimicrobial, anti-biofilm, and synergistic traits of thymol and carvacrol in combat against *Klebsiella*.

**Methods:**

Searching PubMed, Scopus, and Web of Science, we reviewed available evidence on the antibacterial effects of thymol, carvacrol, or combined with other compounds against *Klebsiella* until May 2024*.* Reference checking was performed after the inclusion of studies. Minimum inhibitory concentration (MIC), minimum bactericidal concentration (MBC), fractional inhibitory concentration (FIC), and anti-biofilm activity were gathered, and the MBC/MIC ratio was calculated to assess the bactericidal efficacy.

**Results:**

We retrieved 38 articles out of 2,652 studies screened. The gathered data assessed the anti-microbial activity of thymol, carvacrol, and both compounds in 17, 10, and 11 studies, respectively. The mean (± standard deviation) non-weighted MIC was 475.46 μg/mL (±509.95) out of 60 MIC for thymol and 279.26 μg/mL (±434.38) out of 68 MIC for carvacrol. Thymol and carvacrol showed anti-biofilm activities in the forms of disruption, inhibition, and mass reduction of biofilms. The MBC/MIC ratio was lower than 4 in 45 out of 47 cases, showing high bactericidal efficacy. FIC values were gathered for 68 combinations of thymol and carvacrol with other compounds, and they were mostly synergistic or additive.

**Conclusion:**

Thymol and carvacrol alone or in combination with other compounds, specifically known antibiotics, show great antimicrobial activity.

## 1 Introduction

*Klebsiella pneumoniae* (*K. pneumoniae*), a member of the Enterobacteriaceae family, is a part of the ESKAPE pathogens (*Enterococcus faecium, Staphylococcus aureus, Klebsiella pneumoniae, Acinetobacter baumannii, Pseudomonas aeruginosa,* and *Enterobacter*), known primarily for their antibiotic resistance and association with hospital-acquired infections (Nanayakkara et al., 2021; Ma et al., 2020). Over the years, the ESKAPE pathogens have transformed into multi-drug resistance (MDR) microorganisms. They are now prioritized as a global health threat by the World Health Organization (WHO) due to the mortality, morbidity, and economic burden they cause (Jesudason, 2024). In a systematic review by Ayobami et al., the antibiotic resistance rate of the ESKAPE pathogens in lower and middle-income countries was estimated to be as high as 85.5% for critical antibiotics. They found that the most commonly reported antibiotic resistance was against third-generation-cephalosporins and was particularly among *Escherichia coli* (*E. coli*), *K. pneumoniae*, and *Enterobacter* spp. (Ayobami et al., 2022). According to reports, *K. pneumoniae* resistance to carbapenem rates exceeded 50% in two WHO regions (Zhen et al., 2019). *K. pneumoniae* is isolated from patients with pyogenic liver abscess (Zhang et al., 2019), community-acquired, ventilator-associated, and intensive care unit (ICU)-associated pneumonia (Sharma et al., 2023; Bodmann, 2005), wound infection (Chang et al., 2021), and meningitis (Pu et al., 2023).

The growing emergence of antimicrobial-resistant pathogens has shifted attention to alternative antibacterial agents, including medicinal plants, which have been used since the beginning of humanity (Idris and Nadzir, 2023). According to the WHO, in 2019, antimicrobial resistance (AMR) directly caused 1.27 million deaths, contributed to 4.95 million deaths, and in total, was responsible for 6.22 million deaths globally (Antimicrobial Resistance Collaborators, 2022). Essential oils (EOs), such as *lavender, tea tree,* and *peppermint*, are secretions of herbal plants obtained through fermentation, expression, extraction, or enfleurage. They are used in various industries, including culinary, cosmetics, perfumes, insecticides, and pharmaceuticals (Hoffmann, 2020). These natural products have been meticulously studied for antimicrobial purposes over the years, leading to the identification of several components. One of these components is monoterpenes, which are secondary metabolites found in the EOs of aromatic plants, such as Thymus, Lamiaceae, Origanum, and Lippa peppercorn family (Dehsheikh et al., 2020; Jurevičiūtė et al., 2019; Zhao et al., 2022; Sukmawan et al., 2021). Monoterpenes can be classified into alkaloids, terpenes, flavonoids, phenolic compounds, resins, polypeptides, coumarins, and glucosinolates (Peter et al., 2024). They exhibit antimicrobial, anticancer, antioxidant, and anti-inflammatory activities, making them an interesting field of research (Durugbo, 2013; Sahoo et al., 2021). Thymol and carvacrol are phenolic monoterpenes, approved by the Federal Drug Administration as safe for human consumption (US Food & Drug Administration, 2024). They are considered potent bioactive compounds due to their chemical structure, specifically the presence of the hydroxyl group, which enhances the antibacterial potential of these compounds, and their mechanism of action (Ultee et al., 2002). Furthermore, the antibiofilm activities of thymol and carvacrol have attracted attention to these phenolic monoterpenes in recent years (Campana and Baffone, 2018; Liu et al., 2021). A brief list of plants containing thymol and carvacrol is presented in Table 1. Studies show that they can demonstrate antibacterial properties through biofilm reduction, inhibition of motility, inhibition of membrane-bound adenosine triphosphatases (ATPases) and efflux pumps, and cell wall membrane disruption (Kachur and Suntres, 2020). Their antibacterial role has been commonly studied against *S. aureus*, *Salmonella*, *Shigella*, and *E. coli* (Ngome et al., 2018; Abdelhamid and Yousef, 2021; Heckler et al., 2021; Cid-Pérez et al., 2024). However, given the significant burden *Klebsiella* infections impose on the healthcare system in terms of mortality and morbidity, there was a pressing need for a systematic review study. Our study, therefore, aimed to systematically review the antibacterial activities of thymol and carvacrol against *Klebsiella*, including their bacteriostatic, bactericidal, anti-biofilm, and synergistic effects, offering a potential solution to the growing concern of multi-drug resistance pathogens.

**TABLE 1:** Common plant sources of thymol and carvacrol.

Thymol sources	Carvacrol sources
*Thymus vulgaris*	*Thymus vulgaris*
*Trachyspermum ammi*	*Lepidium flavum*
*Origanum vulgare*	*Origanum vulgare*
*Monarda genera*	*Citrus aurantium bergamia*
*Lippia thymoides Mart. & Schauer*	*Lavanda* *multifidia*
Scrophulariaceae *Euphrasia rostkoviana*	*Monarda didyma*
*Acanthospermum australe*	*Nigella sativa*

## 2 Methods

We used the PICO strategy for formulating research questions. The strategy was based on population (P): *Klebsiella*, Intervention (I): thymol or carvacrol, Control (C): not applicable, and outcome (O): antibacterial effect. This study followed Systematic Review and Meta-Analysis (PRISMA) guidelines (Page et al., 2021).

### 2.1 Search strategy

A comprehensive and systematic search was conducted on databases, including PubMed, Scopus, and Web of Science to identify the relevant articles published until May 2024.

“Thymol,” “carvacrol,” “antibacterial,” “*Klebsiella pneumoniae,*” “*Klebsiella* infections,” “*Klebsiella oxytoca,*” and related keywords were used. Backward and forward citations were tracked by examining the references of the included studies. No restriction on the year of publication was applied.

### 2.2 Study selection and eligibility criteria

Two independent researchers screened the studies by reading titles and abstracts and then full texts using Rayyan, a web-based tool for systematic reviews, and selected relevant studies. Any discrepancies were resolved through consensus between reviewers, and if necessary, a third reviewer made a decision.

### 2.3 Inclusion and exclusion criteria

Original *in vivo* and *in vitro* studies in the English language that reported effects of thymol and carvacrol, simultaneously or independently, in conjunction with other antibacterial agents, were included.

**FIGURE 1:**
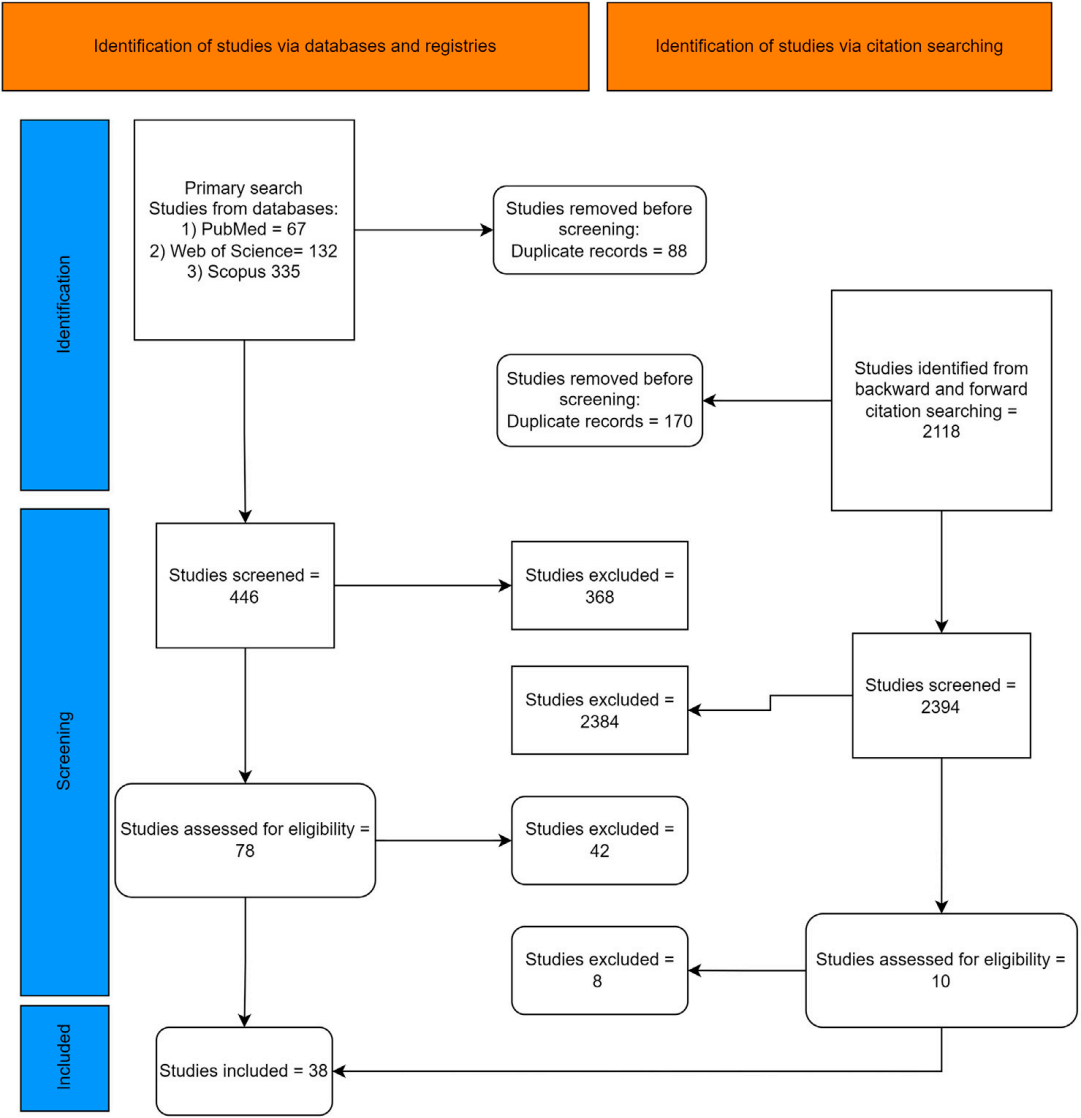
The PRISMA flowchart of the study.

Review articles, editorials/letters, protocols, abstracts, conference articles, meta-analyses, and comments were excluded. Studies without full texts or those involving a mixture of compounds (e.g., herbal essential oils) without the pure forms of thymol and carvacrol were not eligible (Figure 1).

### 2.4 Data extraction

The following data were extracted from the included studies: first author, publication year, country of study, methodology, analyzed compound (thymol or carvacrol or both), *Klebsiella* species, resistance against carbapenems, minimum inhibitory concentration (MIC), minimum bactericidal concentration (MBC), synergistic effects (fractional inhibitory concentration or FIC), and anti-biofilm effects.

Data on antimicrobial resistance to carbapenems were gathered from the studies. If this information was not available, http://ATCC.org was searched using the strain code provided in the study.

### 2.5 Quality assessment

Quality appraisal was conducted by two authors using an adapted version of the Quality Assessment Tool For *In Vitro* Studies (QUIN Tool) (Sheth et al., 2024).

### 2.6 Antibacterial strength

We reported bacteriostatic activity and bactericidal activity of thymol and carvacrol against *Klebsiella* in the form of MIC and MBC, respectively. To compare the antibacterial strength of phenolic compounds, we used the criteria by Taguri et al. (2006) and considered the activity of thymol and carvacrol against *Klebsiella* as strong for MIC <400 μg/mL, moderate for 400 μg/mL < MIC <800 μg/mL, and weak for MIC >800 μg/mL.

Bactericidal efficacy was then calculated using the MBC/MIC ratio, with values less than four considered as good bactericidal efficiency (Bury-Moné, 2014). The methodology and results of studies on anti-biofilm effects were also gathered and presented.

### 2.7 Synergistic activity

We gathered data on the combination of thymol and carvacrol with other compounds and antimicrobials and reported their combination effect using FIC and changes in MIC. The combination effect was considered as synergistic for FIC < 0.5, additive for 0.5 < FIC < 1.0, non-interactive for 1.0 < FIC < 4.0, and antagonistic for FIC > 4.0 (van Vuuren and Viljoen, 2011).

## 3 Results

### 3.1 Search results

Of 2,652 studies screened, 38 (Abdel-halim et al., 2022; Addo et al., 2022; Al-Ani et al., 2015; Alavi and Karimi, 2019; Ndezo et al., 2022; Cordeiro et al., 2020; de Souza et al., 2021; de Souza et al., 2024; Drobac et al., 2017; Gan et al., 2023; Hamoud et al., 2014; Höferl et al., 2009; Huang et al., 2023; Ilić et al., 2017; Iten et al., 2009; Köse, 2022; Kwiatkowski et al., 2022; Liu et al., 2022; Marinelli et al., 2019; Mbese et al., 2022; Mbese et al., 2023; Moghtaderi et al., 2023; Mohammed and Al-Bayati, 2009; Muftah et al., 2020; Bisso et al., 2021; Raei et al., 2017; Rani et al., 2022a; Rani et al., 2022b; Sabour et al., 2019; Salaria et al., 2022; Scandorieiro et al., 2022; Scandorieiro et al., 2023; Tashakor et al., 2024; Yao et al., 2022; Yehia et al., 2024; Zhang et al., 2011; Pormohammad et al., 2022; Choi et al., 2009) studies from 19 different countries were included (Figure 1). All studies showed scores above 70% in quality appraisal using QUIN, indicating a low risk of bias (Supplementary Material S1). A summary of characteristics is available in Table 2.

**TABLE 2:** The characteristics of studies included in the review.

Author / year	Country of study	Method	Thymol / Carvacrol	*Klebsiella*	Carbapenem resistant	MIC value (µg/ml)	MBC value (µg/ml)	MBC / MIC ratio	Key findings
Abdel-halim et al. (2022)	Egypt	In vitro	Thymol	*pneumoniae* (clinical isolates)	Yes	8 strains = 1200^f^ 2 strains = 600^e^	NA	NA	Sub-MIC concentration of thymol sensitized the bacterial cells and weakened the outer membrane
Addo et al. (2022) ^a^	Ghana	In vitro	Thymol	*pneumoniae* NCTC 13438	Yes	Inhibition zone: 2.7mm for 100 thymol	NA	NA	Compared to Thymol, chlorinated nucleus compounds of thymol showed more antibacterial effect against *Klebsiella*.
Al-Ani et al. (2015)	Germany	In vitro	Carvacrol	1) *pneumoniae* ATCC 70060 2) *pneumoniae* ATCC 800877 3) *pneumoniae* ATCC 206436 4) *oxytoca* ATCC 700324	1)No (ESBL) 2) NA 3) NA 4) NA	1) 200^d^ 2) 200^d^ 3) 300^d^ 4) 200^d^	1)300 2)300 3)300 4)200	1) 1.5 2) 1.5 3) 1 4) 1	Carvacrol had MBC/MIC 1-1.5-fold showing strong bactericidal effect against *Klebsiella*.
Alavi and Karimi (2019) ^b^	Iran	In vitro	Thymol	*pneumoniae* K38 (clinical isolates)	NA	60^d^	60	1	Anti-biofilm: In SEM images of morphology changes in *K. pneumoniae* biofilms, star dendrite shape was resulted.
Ndezo et al. (2022)	Cameroon	In vitro	Thymol	*pneumoniae* 1) Kp02 2) Kp03 3) Kp04 4) Kp05 5) Kp55 (clinical isolates)	NA	1) 128^d^ 2) 64^d^ 3) 128^d^ 4) 256^d^ 5) 128^d^	1) 256 2) 512 3) 256 4) 512 5) 512	1) 2 2) 8 3) 2 4) 2 5) 4	Anti-biofilm: Thymol inhibited biofilm formation and disrupted preformed biofilm and induced cell death when combined with streptomycin.
Cordeiro et al. (2020)	Brazil	In vitro	Carvacrol	*pneumoniae* 1)LM-25 2)LM-83 3)LM-173 4)LM-260 5)LM-326 (clinical isolates)	NA	1) 256^d^ 2) 256^d^ 3) 256^d^ 4) 256^d^ 5) 256^d^	NA	NA	Thymol showed additive activity with ceftazidime and cefepime against *Klebsiella*.
de Souza et al. (2021)	Brazil	In vitro and in vivo	Carvacrol	*pneumoniae* 1) bla_kpc-2_ 2) bla_OX1-48_ 3) bla_NDM-1_ 4) bla_CTX-M-8_ 5) altered mgrB (clinical isolates)	Yes (altered mgrB also resistant to Polymyxin B)	1) 130^d^ 2) 130^d^ 3) 260^d^ 4) 130^d^ 5) 130^d^	1) 130 2) 130 3) 260 4) 130 5) 130	1) 1 2) 1 3) 1 4) 1 5) 1	Carvacrol showed MBC/MIC 1-fold against *Klebsiella*. In vivo studies showed a total of growth inhibition within 4 hours (10,25 and 50 mg/kg carvacrol), and a significant reduction of WBC when used with Polymyxin B (2 mg/kg Polymyxin B).
de Souza et al. (2024)	Brazil	In vitro and in vivo	Carvacrol	*pneumoniae* 1)KP-RP03 2)KP-RP05 3)KP-RP10 4)KP-RP12 5)KP-RP20 6)KP-RP25 7)KP-RP29 (clinical isolates)	Yes, also all strains are resistant to Polymyxin B	1) 140^d^ 2) 140^d^ 3) 140^d^ 4) 140^d^ 5) 140^d^ 6) 280^d^ 7) 280^d^	NA	NA	Carvacrol and Polymyxin B combination showed synergistic activity, anti-biofilm formation activity, eliminated all bacterial cells within 2 hours in time-kill assay study, and in vivo study showed all mice treated remained alive compared to 50% death of control group.
Drobac et al. (2017)	Serbia	In vitro	Thymol	*pneumoniae* NCIMB 9111	No	279^d^	NA	NA	Thymol showed antimicrobial activity against Gram- positive, Gram- negative and *Candida* species.
Gan et al. (2023)	Spain	In vitro	Thymol	1) *K. aerogenes* ATCC 13048 2) *pneumoniae* C6	1) No 2) NA	1) 250^d^ 2) 250^d^	1) 250 2) 250	1) 1 2) 1	Thymol showed MBC/MIC 1-fold against *Klebsiella*. Thymol showed additive reaction when combined with chloramphenicol but no interaction with erythromycin, amoxicillin and ampicillin against *Klebsiella*.
Hamoud et al. (2014)	Germany	In vitro	Thymol	*pneumoniae* ATCC 700603	No (ESBL)	500^e^	500	1	Thymol showed MBC/MIC 1-fold against *Klebsiella* Thymol showed moderate activity against Gram- positive and Gram- negative bacteria.
Höferl et al. (2009) ^c^	Bulgaria	In vitro	1) Thymol 2) Carvacrol	*pneumoniae* (clinical isolates)	NA	1) 6 ppm 2) 6 ppm	NA	NA	Thymol and carvacrol showed moderate activity against Gram- positive and Gram- negative bacteria.
Huang et al. (2023)	China	In vitro and in vivo	Thymol gold nanoparticles	*pneumoniae* 1) FK6768 2) FK1913 3) FK8966 4) FK9102 5) FK9283 6) FK3810 (clinical isolates)	1) Resistant 2) Susceptible 3) Resistant 4) Resistant 5) Susceptible 6) Resistant	1) 8^d^ 2) 64^d^ 3) 16^d^ 4) 16^d^ 5) 32^d^ 6) 16^d^ Thymol only 1) ≥256^d^	NA	NA	Thymol had no significant antibacterial activity. Thymol + gold nano particles showed higher antibacterial activity, Anti-biofilm activity via SEM showed reduced bacterial quantity and entity, disintegrated the FK8966 strain cells completely, resulted in protein leakage, and in vivo study resulted in no mice death compared to 90% death in control group.
Ilić et al. (2017)	Serbia	In vitro	Thymol	*pneumoniae* ATCC 700603	No (ESBL)	3123.2^f^	3123.2	1	Thymol combined with streptomycin showed synergistic effect on *Klebsiella* in 10 out of 36 concentration combinations.
Iten et al. (2009)	Germany	In vitro	1) Thymol 2) Carvacrol	*pneumoniae* DSM-Nr.: 681	NA	1) 240^d^ 2) 260^d^	NA	NA	Carvacrol combined with thymol showed almost synergistic activity.
Köse (2022)	Turkey	In vitro	Carvacrol	*pneumoniae* (clinical isolates)	Yes	5 strains = 32^d^ 9 strains = 64^d^ 11 strains = 128^d^	NA	NA	Carvacrol and meropenem showed no bactericidal effect alone, but in combination showed synergistic bactericidal effect against carbapenem resistant *Klebsiella*. This combination also showed serious damage to bacterial cells but was not toxic on vero cells.
Kwiatkowski et al. (2022)	Poland	In vitro	Thymol and Carvacrol	*pneumoniae* 1) NDM-1-producing 2) NDM-1-producing 3) NDM-1-producing 4) BAA-2473 (clinical isolates)	1) Yes 2) Yes 3) Yes 4) No	Thymol/ Carvacrol 1) 780^e^/ 1910^f^ 2) 780^e^/ 1910^f^ 3) 780^e^/ 1910^f^ 4) 780^e^/1910^f^	Thymol/ Carvacrol 1) 1560/ 1910 2) 1560/ 1910 3) 1560/ 1910 4) 1560/ 1910	Thymol/ Carvacrol 1) 2/1 2) 2/1 3) 2/1 4) 2/1	Thymol had a double MBC value compared to MIC and carvacrol had the same MBC value as MIC thus showing good bactericidal activity. Thymol and carvacrol reduced *Klebsiella* biofilm mass in 2 carbapenem-resistant isolates.
Liu et al. (2022)	China	In vitro	Thymol and Carvacrol	*pneumoniae* 1) 208G28 (from chicken) 2) BNCC 102997	NA	Thymol / Carvacrol 1) 198.4^d^/ 241.4^d^ 2) 198.4^d^/ 241.4^d^	NA	NA	Thymol showed the most antibacterial effect and in combination with carvacrol showed better activity.
Marinelli et al. (2019)	Italy	In vitro	Carvacrol	*pneumoniae* ATCC 700603	No (ESBL)	MIC 50 %: 512 MIC 90 %: 512	NA	NA	Highest antibacterial activity against Gram- negative bacteria was seen in water-soluble derivatives of carvacrol
Mbese et al. (2022)	South Africa	In vitro	Carvacrol	*oxytoca* ATCC 8724	NA	20^d^	NA	NA	Study showed that carvacrol and carvacrol hybrids have antibacterial effects against Gram- positive and Gram- negative bacteria.
Mbese et al. (2023) ^2^	South Africa	In vitro	Carvacrol	1) *oxytoca* ATCC 8724 2) *pneumoniae* ATCC 13882	1) NA 2) NA	Inhibition zone: 1) 13 mm 2) 10 mm	NA	NA	Combination of carvacrol with silver nanoparticle gels was ineffective against *Klebsiella*.
Moghtaderi et al. (2023)	Iran	In vitro	Thymol	*pneumonia* ATCC 13883	NA	250^d^	NA	NA	Combination of thymol with noisome and gelatin methacryloyl increased antibacterial activity against *Klebsiella*. Anti-biofilm analysis using crystal violet staining showed 26% decrease in cells.
Mohammed and Al-Bayati (2009)	Iraq	In vitro	Thymol (isolated)	*pneumoniae*	NA	250^d^	NA	NA	Thymol combined with eugenol inhibited the growth of most of the studied bacteria.
Muftah et al. (2020)	Turkey	In vitro	Thymol	*pneumoniae* ATCC 07005	NA	32^d^	NA	NA	Thymol showed antimicrobial activity against Gram- positive, Gram- negative and *Candida* species.
Bisso et al. (2021)	Cameroon	In vitro	Thymol	*Pneumoniae* 1)kp02 2)kp03 3)kp04 4)kp05 5)kp55 (clinical isolates)	NA	1) 128^d^ 2) 64^d^ 3) 128^d^ 4) 256^d^ 5) 128^d^	1) 256 2) 512 3) 256 4) 512 5) 512	1) 2 2) 8 3) 2 4) 2 5) 4	Anti-biofilm: thymol + streptomycin, kanamycin, and amikacin inhibited biofilm formation and showed disperse activity.
Raei et al. (2017) ^3^	Iran	In vitro	Thymol and Carvacrol	*pneumoniae* 1) NDM 2) VIM-1 3) OXA-48 4) KPC	Yes	Thymol / Carvacrol 1) 400^d^/250^d^ 2) 200^d^/125^d^ 3) 200^d^/ 125^d^ 4) 200^d^/ 125^d^	NA	NA	Anti-biofilm: Study showed increasing the concentration of Thymol and carvacrol significantly decreased biofilm formation.
Rani et al. (2022a)	India	In vitro	1) Thymol 2) Carvacrol	*Pneumoniae* ATCC 700603	No (ESBL)	1) 750^e^ 2) 750^e^	1) 1500 2) 1500	1) 2 2) 2	Thymol had MBC/MIC 2-fold against *Klebsiella*. Combination of Thymol or carvacrol with octanoic acid, decanoic acid or lauric acid showed bactericidal activity. Both Thymol and carvacrol had MBC twice the MIC value.
Rani et al. (2022b)	India	In vitro	1) Thymol 2) Carvacrol	*pneumoniae* ATCC 27736	NA	1) 660^e^ 2) 750^e^	1) 1320 2) 750	1) 1 2) 1	Thymol and carvacrol showed bactericidal activity. Combination of Carvacrol with Octanoic acid disrupted cell wall and membrane.
Sabour et al. (2019)	Morocco	In vitro	Thymol	*pneumoniae* CIP 104216	No	780^e^	3130	4	Enhanced antibacterial activity was shown in thymol esters and ethers.
Salaria et al. (2022)	India	In vitro	Thymol	*pneumoniae* MTCC 39	NA	2.5%	NA	NA	Thymol combined with vancomycin or tetracycline showed synergistic effects and an 8-fold increase in effectiveness of antibiotics.
Scandorieiro et al. (2022)	Brazil	In vitro	Thymol and Carvacrol	1) *pneumoniae* ATCC 10031 (reference strain) 2) *pneumoniae* KPC 5795 (clinical isolate)	1) No 2) Yes	Thymol / Carvacrol 1) 250^d^/ 150^d^ 2) 500^e^/ 610^e^	Thymol / Carvacrol 1) 250/310 2) 500/610	Thymol / Carvacrol 1) 1/1 2) 1/1	Thymol and carvacrol had MBC/MIC 1-2 fold and Time- kill curve study showed fast reduction of bacterial cells, thus showing bactericidal activity of thymol and carvacrol.
Scandorieiro et al. (2023)	Brazil	In vitro	Thymol and Carvacrol	KPC-KP 52 (clinical isolates)	Yes	Sessile MIC (pre-formed / formation): Thymol: 310 / 310 Carvacrol: 250 / 310	NA	NA	Anti-biofilm: Thymol and carvacrol combined with silver nanoparticles in SMIC and SEM studies, prevented biofilm formation, disrupted pre-formed biofilm and decreased biofilm activity.
Tashakor et al. (2024)	Iran	In vitro	Thymol	*pneumoniae* 1) ATCC 700603 2) ATCC 13883	1) No (ESBL) 2) NA	1) 625^e^ 2) 625^e^	NA	NA	Thymol encapsulated in Ferula assafoetida gum has increased activity and when combined with imipenem shows synergistic activity.
Yao et al. (2022)	China	In vitro	Thymol	*pneumoniae* 1)FK20 2)FK150 3)FK169 4)FK1342 5)FK1986 6)FK3810 7)FK6663 8)FK6696 (clinical isolates)	1) Yes 2) No 3) Intermediate 4) No 5) No 6) Yes 7) Yes 8) Yes (All colistin resistant)	1) 256^d^ 2) 256^d^ 3) 256^d^ 4) 256^d^ 5) 256^d^ 6) 128^d^ 7) 256^d^ 8) 128^d^	NA	NA	Thymol reduced colistin MIC of all resistant bacteria and Time- kill study showed synergistic activity with colistin. Anti-biofilm: Against colistin resistant *Klebsiella* biofilm formation thymol alone and combined with colistin showed inhibition. In SEM the combination reduced cell numbers, biofilm number and density and bacterial aggregation In vivo study showed higher efficacy in inhibiting colistin resistant klebsiella when thymol was combined with colistin. Thymol increases membrane permeability.
Yehia et al. (2024) ^3^	Egypt	In vitro	Carvacrol	*oxytoca* (from chicken)	Intermediate	156^d^	NA	NA	Carvacrol and carvacrol-loaded invasomes have antibacterial activity.
Zhang et al. (2011)	China	In vitro	1) Thymol 2) Carvacrol	*oxytoca* (from animal feed)	No	1) 1250 mM/mL 2) 2500 mM/mL	NA	NA	Thymol and carvacrol combined with nitrofurantoin and ampicillin have synergistic activity.
Pormohammad et al. (2022)	Canada	In vitro and in vivo	Thymol and Carvacrol	1) *pneumoniae* ATCC 11296 2) *pneumoniae* ATCC 11296 3) *pneumoniae* ATCC 11296	NA	Thymol / Carvacrol 1) 30^d^/ 147^d^ 2) 62.5^d^/ 125^d^ 3) 31^d^/ 250^d^	Thymol / Carvacrol 1) 30 / 147 2) 62 / 125 3) 64 / 250	Thymol / Carvacrol 1) 1/1 2) 1/1 3) 2/1	Anti-biofilm: Thymol and carvacrol showed bactericidal and antibiofilm activity. Out of 15 plant based natural compounds studied, thymol was the most effective against *Klebsiella*. In vitro studies showed carvacrol increases growth, motility and regeneration of *C. elegans* thus being even healthy.
Choi et al. (2009) ^b^	Korea	In vitro	Carvacrol	*oxytoca*	NA (nalidixic acid resistant)	125^d^	NA	NA	Nalidixic acid and carvacrol have indifferent effect when combined against nalidixic acid-resistant *Klebsiella*.

^a^ Well diffusion.

^b^ Disc diffusion.

^c^ Agar dilution.

^d^ = strong.

e = moderate.

f = weak, ESBL, extended spectrum beta-lactamase.

NA, not available; SEM, scanning electron microscopy; SMIC, sessile minimum inhibitory concentration.

### 3.2 Anti-microbial and anti-biofilm effects

Data on the anti-microbial activity of thymol (Abdel-halim et al., 2022; Addo et al., 2022; Alavi and Karimi, 2019; Ndezo et al., 2022; Drobac et al., 2017; Gan et al., 2023; Hamoud et al., 2014; Huang et al., 2023; Ilić et al., 2017; Moghtaderi et al., 2023; Mohammed and Al-Bayati, 2009; Muftah et al., 2020; Bisso et al., 2021; Sabour et al., 2019; Salaria et al., 2022; Tashakor et al., 2024; Yao et al., 2022), carvacrol (Al-Ani et al., 2015; Cordeiro et al., 2020; de Souza et al., 2021; de Souza et al., 2024; Köse, 2022; Marinelli et al., 2019; Mbese et al., 2022; Mbese et al., 2023; Yehia et al., 2024; Choi et al., 2009) and both compounds (Höferl et al., 2009; Iten et al., 2009; Kwiatkowski et al., 2022; Liu et al., 2022; Raei et al., 2017; Rani et al., 2022a; Rani et al., 2022b; Scandorieiro et al., 2022; Scandorieiro et al., 2023; Zhang et al., 2011; Pormohammad et al., 2022) were obtained from 17, 10, and 11 studies, respectively. All studies used purchased pure forms of thymol and carvacrol, except the study by Mohammed and Al-Bayati (2009), which isolated thymol from essential oils.

All studies assessed anti-bacterial activity against *K. pneumoniae*, except four against *K. oxytoca* (Mbese et al., 2022; Yehia et al., 2024; Zhang et al., 2011; Choi et al., 2009). Two studies compared *K. pneumoniae* and *K. oxytoca* (Al-Ani et al., 2015; Mbese et al., 2023)*,* and one compared *K. aerogenes* and *K. pneumonia* (Gan et al., 2023).

Regarding the sources of isolates, 14 studies used clinical isolates (Abdel-halim et al., 2022; Alavi and Karimi, 2019; Ndezo et al., 2022; Cordeiro et al., 2020; de Souza et al., 2021; de Souza et al., 2024; Höferl et al., 2009; Huang et al., 2023; Köse, 2022; Kwiatkowski et al., 2022; Bisso et al., 2021; Scandorieiro et al., 2022; Scandorieiro et al., 2023; Yao et al., 2022), 2 used isolates derived from chicken broilers (Liu et al., 2022; Yehia et al., 2024), 1 from animal feed (Zhang et al., 2011), and others purchased reference strains.

Regarding the availability of MIC data, 37 MIC were available for reference strains, 84 for clinical stains, 5 for strains derived from chicken broiler and 2 for strains from animal feed. The values of non-weighted MIC mean (median) were calculated as follows: for reference strains: 464.65 (250) µg/mL for thymol and 257 (200) µg/mL for carvacrol, for clinical strains: 505.17 (256) µg/mL for thymol and 288.83 (128) µg/mL for carvacrol, for chicken broiler strains: 198.40 (198.40) µg/mL for thymol and 212.93 (241.40) µg/mL for carvacrol, and for animal feed strains: 187 (187) µg/mL for thymol and 375 (375) µg/mL for carvacrol.

Regarding the methods used for MIC assessment, all studies used broth dilution except 1 (Addo et al., 2022), which used well diffusion, 3 (Alavi and Karimi, 2019; Mbese et al., 2023; Choi et al., 2009), which used disc diffusion, and 3 (Höferl et al., 2009; Raei et al., 2017; Yehia et al., 2024), which used agar dilution.

**FIGURE 2:**
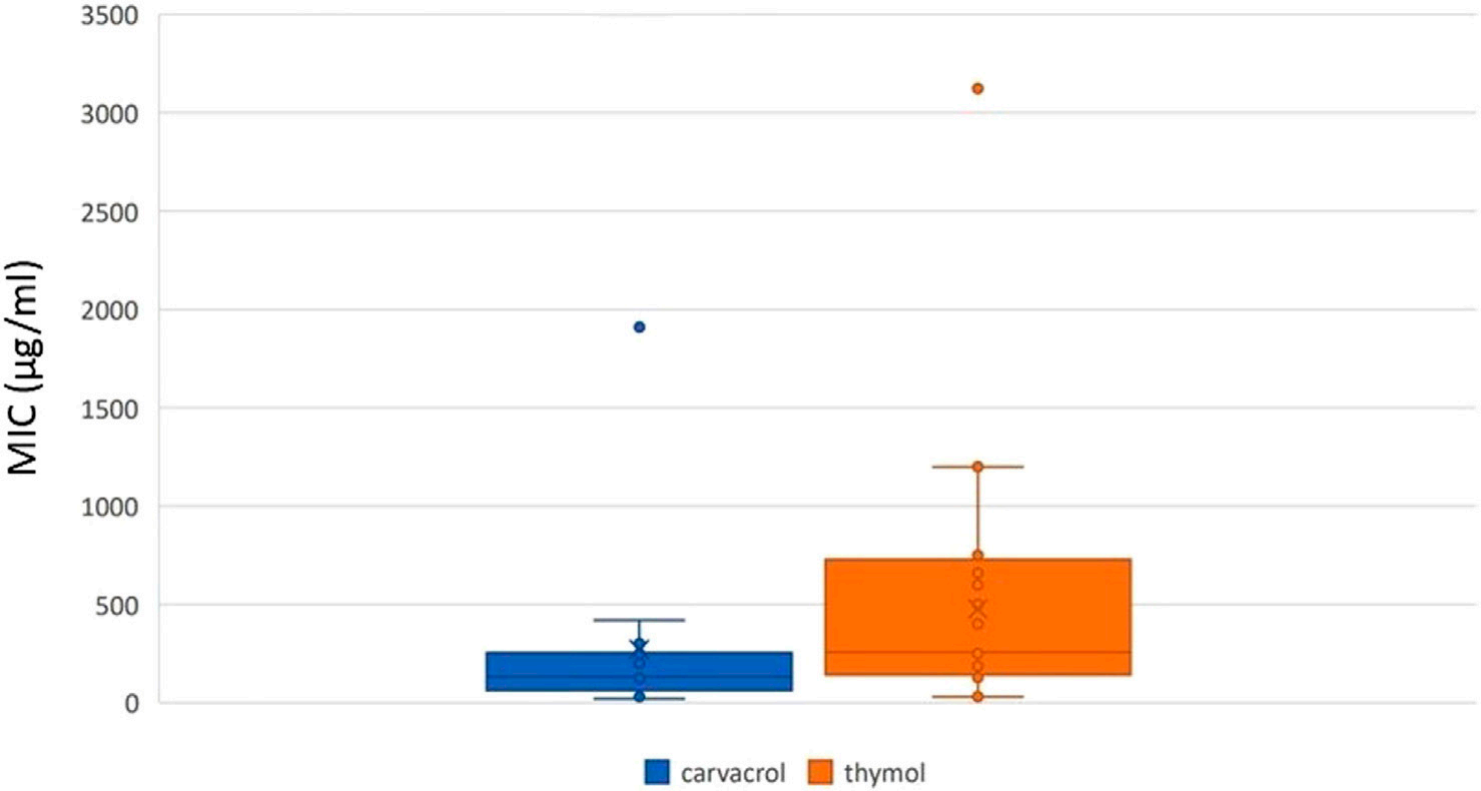
MIC for thymol and carvacrol.

Regarding the assessment of antibacterial activity, two studies provided inhibition zone diameter (Addo et al., 2022; Mbese et al., 2023), one provided sessile MIC calculated for anti-biofilm activity (Scandorieiro et al., 2023), two studies did not provide MIC in µg/mL (Salaria et al., 2022; Zhang et al., 2011), and one provided MIC 50% and 90% (Marinelli et al., 2019). A total of 128 MIC in µg/mL were gathered, with 60 MIC reported for thymol, ranging from 30 μg/mL to 3,123 μg/mL, and 68 MIC reported for carvacrol, ranging from 32 μg/mL to 1910 μg/mL. Additionally, 99 MIC values were lower than 400 μg/mL and considered strong, while 16 were moderate and 13 were weak. The mean (± standard deviation, median) non-weighted MIC was 475.46 μg/mL (±509.95, 256 μg/mL) for thymol and 279.26 μg/mL (±434.38, 130 μg/mL) for carvacrol (Figure 2), with carvacrol MIC being significantly lower than thymol MIC (P = 0.022).

Carbapenem-resistant *Klebsiella* was reported in 11 studies (Abdel-halim et al., 2022; Addo et al., 2022; de Souza et al., 2021; de Souza et al., 2024; Huang et al., 2023; Köse, 2022; Kwiatkowski et al., 2022; Raei et al., 2017; Scandorieiro et al., 2022; Scandorieiro et al., 2023; Yao et al., 2022), with 68 MIC ranging from 32 μg/mL to 1910 μg/mL. The mean (± standard deviation, median) non-weighted MIC for carbapenem-resistant *Klebsiella* was 681.04 μg/mL (±216.61, 600 μg/mL) for thymol and 247.76 μg/mL (±68.44, 128 μg/mL) for carvacrol.

The anti-biofilm effect against *Klebsiella* was reported in 11 studies, with 6 studies assessing thymol (Alavi and Karimi, 2019; Ndezo et al., 2022; Huang et al., 2023; Moghtaderi et al., 2023; Bisso et al., 2021; Yao et al., 2022), 1 assessing carvacrol (de Souza et al., 2024), and 4 examining both (Kwiatkowski et al., 2022; Raei et al., 2017; Scandorieiro et al., 2023; Pormohammad et al., 2022). The studies showed that thymol and carvacrol can have multiple anti-biofilm mechanisms against *Klebsiella*, including changing the cell morphology, inhibition of biofilm formation, disruption of preformed biofilm, reduction of bacterial mass, and synergistic activity with antibiotics.

### 3.3 Bactericidal effects

To evaluate the bactericidal efficacy of thymol and carvacrol, we calculated the MBC/MIC ratio in studies reporting MBC. Of the 14 studies providing MBC values (Al-Ani et al., 2015; Alavi and Karimi, 2019; Ndezo et al., 2022; de Souza et al., 2021; Gan et al., 2023; Hamoud et al., 2014; Ilić et al., 2017; Kwiatkowski et al., 2022; Bisso et al., 2021; Rani et al., 2022a; Rani et al., 2022b; Sabour et al., 2019; Scandorieiro et al., 2022; Pormohammad et al., 2022), a total of 47 MBC/MIC ratios were calculated. Of these, 45 ratios were four or less, and only two ratios from two studies (Ndezo et al., 2022; Bisso et al., 2021) were higher, indicating substantial bactericidal activities for thymol and carvacrol. These two ratios, both equal to eight, were against clinical isolates.

### 3.4 Combination effects

The anti-bacterial combination effects of thymol and carvacrol with other compounds (Table 3), were assessed in 19 studies (Abdel-halim et al., 2022; Alavi and Karimi, 2019; Ndezo et al., 2022; Cordeiro et al., 2020; de Souza et al., 2024; Gan et al., 2023; Huang et al., 2023; Ilić et al., 2017; Köse, 2022; Bisso et al., 2021; Rani et al., 2022a; Rani et al., 2022b; Salaria et al., 2022; Scandorieiro et al., 2022; Scandorieiro et al., 2023; Tashakor et al., 2024; Yao et al., 2022; Zhang et al., 2011; Choi et al., 2009), with 10 studies assessing thymol (Abdel-halim et al., 2022; Alavi and Karimi, 2019; Ndezo et al., 2022; Gan et al., 2023; Huang et al., 2023; Ilić et al., 2017; Salaria et al., 2022; Tashakor et al., 2024; Yao et al., 2022), four assessing carvacrol (Cordeiro et al., 2020; de Souza et al., 2024; Köse, 2022; Choi et al., 2009), and 5 assessing both (Rani et al., 2022a; Rani et al., 2022b; Scandorieiro et al., 2022; Scandorieiro et al., 2023; Zhang et al., 2011).

**TABLE 3:** The combination activity of thymol and carvacrol with other compounds.

Author / year	Thymol / Carvacrol	*Klebsiella*	Combination	MIC reduction for antibiotic	FIC	Combination effect
Abdel Halim et al. (2022)	Thymol (300 µg/mL)	*pneumoniae* ^e^	Meropenem	2-fold reduction in meropenem MIC against 5 out of 8 *Klebsiella* strains	NA	Sub-inhibitory concentration of thymol when combined with meropenem and celastrol increase meropenem effect
		*pneumoniae* ^e^	Celastrol + Meropenem	4 to more than 64-fold reduction of meropenem MIC	NA	
Alavi and Karimi (2019) ^d^	Thymol	*pneumoniae* K38	AgNO3and CuSO4 metal nanoparticle	Combination increased thymol MIC from 60 to 80 µg/mL	NA	Although MIC increased, protein leakage was increased when thymol was combined with CuSO4, showing increased bactericidal activity
Bisso et. al. 2022^d^	Thymol	*pneumoniae* 1) Kp02 2) Kp03 3) Kp04 4) Kp05 5) Kp55	lactic-co-glycolic acid (PLGA) nanoparticles	1) 32-fold 2) 64-fold 3) 16-fold 4) 32-fold 5) 64-fold Thymol MIC reduction	NA	Apart from MIC, antibiofilm effects of streptomycin was assessed, and shown to increase when combined with thymol
Cordeiro et al (2020)	Carvacrol	*pneumoniae* 1)LM-25 2)LM-83 3)LM-173 4)LM-260 5)LM-326	Ceftazidime	NA	1) 0.75 2) 0.75 3) 0.75 4) 0.75 5) 0.75	Carvacrol showed additive association when combined with cefepime or ceftazidime
			Cefepime	NA	1)0.62 2)0.56 3)0.62 4)0.56 5)0.75	
De Souza et al (2024)	Carvacrol	*pneumoniae* ^e^ (Polymyxin resistant)	Polymyxin B	NA	Lowest FIC: 0.125	Thymol + polymyxin B had synergistic association. This combination also had bactericidal effect against polymyxin resistant *Klebsiella*.
Gan et al (2023)	Thymol	*aerogenes* ATCC 13048	1) Chloramphenicol 2) Erythromycin	1) 2-fold 2) No change	1) 0.56 2) 2	Thymol had additive activity when combined with chloramphenicol
		*pneumoniae* C6	1) Amoxycillin 2) Ampicillin 3) Erythromycin	1) No change 2) No change 3) No change	1) 2 2) 2 3) 2	Thymol had no combined effect against *K. pneumoniae* when combined with other antibiotics.
Huang et. al. 2017^a^	Thymol silver nanoparticles	*pneumoniae* FK6768^e^	Gold nanoparticles	More than 32-fold reduction in thymol MIC	NA	Thymol + gold nanoparticles showed greater bactericidal activity.
Ilić et al (2017)	Thymol	*pneumoniae* ATCC 700603	1) Streptomycin 2) Geraniol	Highest: 1)6.66-fold^a^ 2)5.8-fold^a^	Lowest: 1) 0.65 2)0.87	Thymol had additive activity with streptomycin and geraniol.
Köse et al (2022)	Carvacrol	*pneumoniae* ^e^	Meropenem	Highest: 8-Fold^a^	Lowest: 0.5	Carvacrol had synergistic activity with meropenem against resistant *Klebsiella*.
Ndezo et. al. 2021^b^	Thymol	*pneumoniae* 1)kp55 2)kp02 3)kp03 4)kp04 5)kp05	Streptomycin	1) 64-fold 2) 16-fold 3) 16-fold 4) 16-fold 5) 64-fold	1) 0.14 2) 0.13 3) 0.19 4) 0.13 5) 0.27	Thymol showed mostly synergistic activity in combination with streptomycin, amikacin, and kanamycin against biofilm formation of *Klebsiella*.
			Amikacin	1) 16-fold 2) 8-fold 3) 2-fold 4) 4-fold 5) 2-fold	1) 0.13 2) 0.25 3) 1 4) 0.5 5)1.5	
			Kanamycin	1) 4-fold 2) 16-fold 3) 1-fold 4) 32-fold 5) 4-fold	1) 0.28 2) 0.31 3) 1.06 4) 0.16 5) 1.25	
Rani et al. (2022a) ^a^	Thymol	*pneumoniae* ATCC 700603	1) Octanoic Acid 2) Decanoic Acid 3) Lauric Acid	1) 8.33-fold^a^ 2) 3.94-fold^a^ 3) 3.94-fold^a^	1) 0.56 2) 0.75 3) 0.2	Combination of thymol and carvacrol with octanoic and decanoic acids has additive effect against *Klebsiella*.
	Carvacrol		1) Octanoic Acid 2) Decanoic Acid 3) Lauric Acid	1) 1.97-fold^a^ 2) 1.97-fold^a^ 3) 1.97-fold^a^	1) 0.63 2) 0.75 3) 2	
Rani et. al. 2023^a^	Thymol	*pneumoniae* ATCC 27736	1) Octanoic Acid 2) Decanoic Acid 3) Lauric Acid	1) 1.97-fold^a^ 2) 1.97-fold^a^ 3) 1.97-fold^a^	1) 0.75 2) 1.5 3) 1.5	Thymol + octanoic acid had additive effect.
	Carvacrol		1) Octanoic Acid 2) Decanoic Acid 3) Lauric Acid	1) 1.97-fold^a^ 2) 3.94-fold^a^ 3) 1.97-fold^a^	1) 0.88 2) 0.75 3) 0.76	Carvacrol + octanoic, decanoic, and lauric acids was additive.
Salaria et al. (2022)	Thymol	*pneumoniae* MTCC 39	1) Tetracycline 2) Vancomycin	Highest: 1) 8-fold 2) 8-fold	Lowest: 1) 0.16 2) 0.25	Thymol showed synergistic activity when combined with vancomycin or tetracycline.
Scandorieiro et al. (2022) ^a^	Thymol	1) *pneumoniae* ATCC 10031 2) *pneumoniae* KPC 5795^e^	Silver biogenic nanoparticles	1) 8-fold 2) 8-fold	1) 0.62 2) 0.62	Thymol and carvacrol combined biogenic with each other or silver nanoparticles shows additive activity.
	Carvacrol			1) 2-fold 2) 4-fold	1) 1 2)0.75	
	Carvacrol +Thymol		Carvacrol/ Thymol	1) 8-fold/ 2-fold 2) 2-fold/ 8-fold	1) 0.62 2) 0.62	
Scandorieiro et al. (2023) ^c^	Thymol	*pneumoniae* KPC^e^	Silver biogenic nanoparticles	2-fold	NA	Thymol and carvacrol combined biogenic silver nanoparticles show additive effect.
	Carvacrol			2-fold	NA	
	Carvacrol +Thymol		Carvacrol / Thymol	No change / No change	NA	Thymol combined with carvacrol shows no additive activity.
Tashakor et al. (2024)	Thymol capsulated in Ferula assafoetida gum (AFG)	*pneumoniae*	Imipenem	8-fold^a^	0.375	Thymol with Ferula assafoetida gum shows synergistic activity with imipenem.
Yao et al. (2022)	Thymol	*pneumoniae* (colistin resistant) 1)FK20^e^ 2)FK150 3)FK169 4)FK1913 5)FK1986 6)FK3810^e^ 7)FK6663^e^ 8)FK6696^e^	Colistin	1) 32-fold 2) 32-fold 3) ≥64-fold 4) ≥256-fold 5) 128-fold 6) ≥256-fold 7) 256-fold 8) ≥256-fold	1) 0.53 2) 0.16 3) 0.14 4) 0.25 5) 0.13 6) 0.13 7) 0.25 8) 0.50	Thymol + colistin shows mostly synergistic activity against colistin-resistant *Klebsiella*.
Zhang et. al. 2021	Thymol	*oxytoca*	1) Nitrofurantoin 2) Ampicillin	NA	1)0.55 2)0.75	Thymol showed additive activity with nitrofurantoin and ampicillin.
	Carvacrol		1) Nitrofurantoin 2) Ampicillin	NA	1)0.15 2)0.375	Carvacrol showed synergistic activity with nitrofurantoin and ampicillin.
Choi et al. (2009)	Carvacrol	*oxytoca* (Nalidixic acid resistant)	Nalidixic acid	No change	1.5	Carvacrol had no effect on nalidixic acid MIC against resistant *Klebsiella*.

^a^ Calculated using available data.

^b^ Antibiofilm activity measured using Mean biofilm inhibitory concentration (MBIC).

^c^ sessile MIC against biofilm formation.

^d^ MIC change compared to thymol MIC because the combination was not with a common antimicrobial.

^e^ carbapenem resistant.

NA = not available.

The lowest FIC value for each *Klebsiella* strain and compound, in combination with thymol or carvacrol, was gathered, resulting in 68 FIC, as shown in Table 3. We found that 25 combinations were synergistic, 32 were additive, and 11 were non-interactive. The change in antibiotic MIC is also available, ranging from no change for erythromycin, amoxicillin, and ampicillin when combined with thymol (Gan et al., 2023), and for nalidixic acid when combined with carvacrol (Choi et al., 2009) to more than 256-fold antibiotic MIC reduction for colistin when combined with thymol against colistin-resistant *Klebsiella* (Yao et al., 2022). This substantial reduction in colistin MIC was possibly due to the increased permeability of the *Klebsiella* outer membrane in the presence of thymol (Yao et al., 2022). Overall, thymol was assessed in more combinations and showed more synergistic activities with other compounds than carvacrol.

When combined with known antimicrobial agents (i.e., meropenem, ceftazidime, cefepime, polymyxin B, chloramphenicol, erythromycin, amoxicillin, ampicillin, streptomycin, amikacin, kanamycin, tetracycline, vancomycin, imipenem, colistin, nitrofurantoin, and nalidixic acid), pure thymol showed FIC<1 or at least a 2-fold reduction in the antimicrobial agent MIC for 35 out of 42 combinations (83.3%), while pure carvacrol showed FIC<1 or at least a 2-fold reduction in the antimicrobial agent MIC for 14 out of 15 combinations (93.3%).

## 4 Discussion

In this systematic review, we aimed to provide new insights into the activities of two terpenoids, carvacrol and its isomer thymol, against an ESKAPE pathogen, *Klebsiella*. We gathered data regarding MIC, MBC, MBC/MIC ratio, anti-biofilm, and the combination effect with antibiotics in order to appraise antimicrobial activities of these two compounds.

The MIC values, used as a measure of antimicrobial inhibition, were collected and found to vary widely. In a systematic review by Truong et al. investigating the antibacterial effects of *Lavender* EOs against methicillin-resistant *S*. *aureus*, inconsistent results were noticed due to variability in materials, bacterial strains, and methodology (Truong and Mudgil, 2023). Similarly, we observed variability in *Klebsiella* strain, type of *Klebsiella* sampling, antimicrobial resistance pattern, and methodology of MIC measurement. Nevertheless, results indicated strong bacteriostatic activity (104 out of 132 MIC, 78.8%) for both thymol (44 out of 65 strong, 67.7%) and carvacrol (60 out of 67 strong, 89.5%).

Additionally, we observed variability in MBC values. To deal with this variability in results, we calculated the MBC/MIC ratios, and found that 45 out of 47 ratios were lower than four, showing the homogeneity in bactericidal effect and high bactericidal efficacy of both thymol and carvacrol. The bactericidal activity of thymol and carvacrol was previously demonstrated against *S*. *aureus* (Zhou et al., 2019; Rúa et al., 2011)*, Shigella flexnri* (Ngome et al., 2018)*, Actinobacillus pleuropneumoniae* (Wang et al., 2017)*, A. baumannii* (Hassannejad et al., 2019)*, Staphylococcus pseudintermedius, Proteus mirabilis,* and *P*. *aeruginosa* (Sim et al., 2019).

The antibacterial activities of EOs against *Klebsiella* were previously demonstrated for *Monarda didyma* (Chen et al., 2023)*, Satureja nabateorum* (Al-Maharik and Jaradat, 2021), and *Althaea officinalis* (Arab et al., 2023), which constituted mostly of thymol (69.75%, 46.07%, 58.91%, respectively) and for *Lavandula coronopifolia* (Ait Said et al., 2015)*, Thymus capitatus* (Ben Selma et al., 2024)*,* and *Satureja spicigera* (Eftekhar et al., 2009)*,* which constituted mostly of carvacrol (48.9%, 69.28%, 53.74%, respectively). The antibacterial activities of these Eos against *Klebsiella* can therefore be attributed partly to thymol and carvacrol.

The anti-biofilm activity of antimicrobials is crucial in combating *K. pneumoniae*, especially considering the increased risk of infection when medical devices are present (Vuotto et al., 2017). Our collected data showed the anti-biofilm activity of thymol and carvacrol against biofilm formation and pre-formed biofilms. The anti-biofilm activity of thymol and carvacrol was previously demonstrated against *S*. *aureus* and *P*. *aeruginosa* (Walczak et al., 2021). It was also reported against carbapenem-resistant Gram-negative bacilli, such as *Klebsiella*, *Pseudomonas*, and *Acinetobacter* by Raei et al. (2017).

Our study demonstrated antibacterial activity against carbapenem-resistant *Klebsiella*, with strong activity observed in 53 out of 78 available MIC. This activity was not restricted to *Klebsiella*; it also extended to other resistant bacteria, such as *Pseudomonas* and *Acinetobacter* (Raei et al., 2017). Furthermore, the activity was not limited to resistance to carbapenems; it also included resistance to polymyxin B (de Souza et al., 2021; de Souza et al., 2024), nalidixic acid (Choi et al., 2009), colistin (Yao et al., 2022), and ESBL (Al-Ani et al., 2015; Hamoud et al., 2014; Ilić et al., 2017; Marinelli et al., 2019; Rani et al., 2022a; Tashakor et al., 2024). Additionally, in an *in vivo* study using a pneumonic mouse model, Hassannejad et al. illustrated the antibacterial activities of thymol, carvacrol, and *Zataria multiflora boiss* extract, the major constituents of which are thymol and carvacrol, against colistin-resistant *A. baumannii* (Hassannejad et al., 2019).

Interestingly, these two compounds not only demonstrated significant antibacterial activity alone but also when combined with a range of antibiotics, showed additive to synergistic activities. This property can be substantially beneficial, especially against *K*. *pneumoniae* resistant to carbapenems, polymyxin B, and colistin, where the choice of treatment becomes complicated (Ardebili et al., 2023). In our study, we demonstrated not only the synergistic activities of thymol and carvacrol with meropenem (FIC = 0.5) but also a reduction in meropenem MIC when combined with these two compounds against carbapenem-resistant *K. pneumoniae* (Abdel-halim et al., 2022; Köse, 2022). The same results were also available for colistin against colistin-resistant *K*. *pneumoniae* (Yao et al., 2022) and for polymyxin B against polymyxin B*-*resistant *K*. *pneumoniae* (de Souza et al., 2024). This synergistic activity of antibiotics with thymol and carvacrol could be due to their ability to increase bacterial cell wall permeability and cause disruption (Xu et al., 2008). This activity is maintained by permeability to hydrogen and potassium ions through lipid layer destabilization, decrease in elasticity, and increase in fluidity, and by interaction with bacterial proteins (Kowalczyk et al., 2020). These factors may allow the combined antibacterial compound to affect the resistant bacteria.

According to our results, carvacrol exhibited a lower MIC and better synergistic activity. Additionally, previous clinical trials showed the use of carvacrol in patients with asthma (Ghorani et al., 2021a) and veterans exposed to sulfur mustard (Khazdair and Boskabady, 2019). Moreover, a phase I clinical study assessed carvacrol in healthy patients and showed safety and tolerability when carvacrol was used in 1 and 2 mg/kg/day doses (Ghorani et al., 2021b). Therefore, carvacrol seems to be a better candidate for use as an antibacterial agent. The mechanisms of action of carvacrol and thymol are speculated to involve disrupting membrane integrity by integrating into its lipid fragments, depleting the cell of its ATPs and intracellular materials, and thus causing cellular death (Trombetta et al., 2005).

Notably, using thymol and carvacrol as antibacterial agents has some limitations due to their high vaporization and volatility (Escobar et al., 2020). In addition, the low oxidation rate of thymol requires the use of a catalyst to enhance oxidation, which is a common degradation method (Gabrič et al., 2022; Günay et al., 2016). Moreover, carvacrol exhibits low stability, low water solubility, and high sensitivity to the acidity of the digestive system (Günay et al., 2016; Mączka et al., 2023).

Although one of the objectives of this study was to assess the effects of thymol and carvacrol on antimicrobial-resistant *Klebsiella*, many of the included studies did not provide the resistance pattern of the *Klebsiella* strains studied. Also, MIC values were not reported with ranges or standard deviations, preventing us from conducting a meta-analysis. For further research, we recommend reporting all MIC values with standard deviations and providing the resistance pattern of all bacterial strains.

## 5 Conclusion

The results of this systematic review show that thymol and carvacrol have strong bacteriostatic activity and high bactericidal efficacy. They also exhibit anti-biofilm activities and additive to synergistic combination effects with other compounds against *Klebsiella*. Therefore, thymol and, especially, carvacrol possess great potential for future studies on antimicrobial resistance. However, their inherent limitations must be considered.

## Data Availability

The original contributions presented in the study are included in the article/Supplementary Material, further inquiries can be directed to the corresponding author.
