# Supplementary material for: Thymol and carvacrol against Klebsiella: anti-bacterial, anti-biofilm, and synergistic activities—a systematic review
Source: Front Pharmacol. 2024 Oct 24;15:1487083. doi: 10.3389/fphar.2024.1487083 (PMC11540684; doi:10.3389/fphar.2024.1487083)
Supplement: Supplementary file 1 [file Table1.DOCX]

| study | year | Q1 | Q2 | Q3 | Q4 | Q5 | Q6 | Q7 | Q8 | Q9 | Q10 | Q11 | Q12 | Total |
| --- | --- | --- | --- | --- | --- | --- | --- | --- | --- | --- | --- | --- | --- | --- |
| Abdel Halim | **2022** | 2 | NA | 2 | 2 | 1 | NA | NA | 2 | NA | NA | 2 | 2 | 13 |
| Addo | **2022** | 2 | NA | 2 | 2 | 2 | NA | NA | 1 | NA | NA | 2 | 1 | 12 |
| Al-ani | **2015** | 2 | NA | 2 | 2 | 2 | NA | NA | 2 | NA | NA | 2 | 1 | 13 |
| Alavi | **2019** | 2 | NA | 2 | 2 | 1 | NA | NA | 2 | NA | NA | 2 | 1 | 12 |
| Bisso | **2022** | 2 | NA | 2 | 2 | 2 | NA | NA | 2 | NA | NA | 2 | 1 | 13 |
| Cordeiro | **2020** | 2 | NA | 2 | 2 | 2 | NA | NA | 2 | NA | NA | 2 | 1 | 13 |
| De Souza | **2021** | 2 | NA | 2 | 2 | 1 | NA | NA | 2 | NA | NA | 2 | 1 | 12 |
| De Souza | **2023** | 2 | NA | 2 | 2 | 2 | NA | NA | 2 | NA | NA | 2 | 1 | 13 |
| Doughish | **2023** | 2 | NA | 2 | 2 | 1 | NA | NA | 1 | NA | NA | 2 | 1 | 11 |
| Drobac | **2017** | 2 | NA | 2 | 2 | 2 | NA | NA | 2 | NA | NA | 2 | 1 | 13 |
| Gan | **2023** | 2 | NA | 2 | 2 | 1 | NA | NA | 2 | NA | NA | 2 | 1 | 12 |
| Hamoud | **2014** | 2 | NA | 2 | 2 | 2 | NA | NA | 2 | NA | NA | 2 | 1 | 13 |
| Höferl | **2009** | 2 | NA | 2 | 1 | 1 | NA | NA | 1 | NA | NA | 2 | 1 | 10 |
| Huang | **2023** | 2 | NA | 2 | 2 | 1 | NA | NA | 2 | NA | NA | 2 | 1 | 12 |
| Ilić | **2017** | 2 | NA | 2 | 2 | 1 | NA | NA | 2 | NA | NA | 2 | 1 | 12 |
| Iten | **2009** | 2 | NA | 2 | 2 | 1 | NA | NA | 2 | NA | NA | 2 | 1 | 12 |
| Köse | **2022** | 2 | NA | 2 | 1 | 2 | NA | NA | 2 | NA | NA | 2 | 2 | 13 |
| Kwiatkowski | **2022** | 2 | NA | 2 | 2 | 2 | NA | NA | 2 | NA | NA | 2 | 2 | 14 |
| Liu | **2022** | 2 | NA | 2 | 2 | 1 | NA | NA | 2 | NA | NA | 2 | 1 | 12 |
| Marinelli | **2019** | 2 | NA | 2 | 2 | 2 | NA | NA | 1 | NA | NA | 2 | 2 | 13 |
| Mbese | **2022** | 2 | NA | 2 | 2 | 2 | NA | NA | 2 | NA | NA | 2 | 1 | 13 |
| Mbese | **2024** | 2 | NA | 2 | 2 | 2 | NA | NA | 1 | NA | NA | 2 | 1 | 12 |
| Moghtaderi | **2023** | 2 | NA | 2 | 2 | 2 | NA | NA | 2 | NA | NA | 2 | 1 | 13 |
| Mohammed | **2009** | 2 | NA | 2 | 1 | 1 | NA | NA | 2 | NA | NA | 2 | 1 | 11 |
| Muftah | **2020** | 2 | NA | 2 | 2 | 1 | NA | NA | 2 | NA | NA | 2 | 2 | 13 |
| Ndezo | **2021** | 2 | NA | 2 | 2 | 2 | NA | NA | 2 | NA | NA | 2 | 1 | 13 |
| Raei | **2017** | 2 | NA | 2 | 2 | 1 | NA | NA | 2 | NA | NA | 2 | 1 | 12 |
| Rani | **2022** | 2 | NA | 2 | 2 | 2 | NA | NA | 2 | NA | NA | 2 | 2 | 14 |
| Rani | **2023** | 2 | NA | 2 | 2 | 2 | NA | NA | 2 | NA | NA | 2 | 2 | 14 |
| Sabour | **2019** | 2 | NA | 2 | 2 | 1 | NA | NA | 2 | NA | NA | 2 | 2 | 13 |
| Salaria | **2022** | 2 | NA | 2 | 2 | 1 | NA | NA | 1 | NA | NA | 2 | 1 | 11 |
| Scandoireiro | **2022** | 2 | NA | 2 | 2 | 2 | NA | NA | 2 | NA | NA | 2 | 1 | 13 |
| Scandoireiro | **2023** | 2 | NA | 2 | 2 | 1 | NA | NA | 2 | NA | NA | 2 | 1 | 12 |
| Tashakor | **2024** | 2 | NA | 2 | 2 | 1 | NA | NA | 2 | NA | NA | 2 | 1 | 12 |
| Yao | **2022** | 2 | NA | 2 | 2 | 2 | NA | NA | 2 | NA | NA | 2 | 1 | 13 |
| Yehia | **2024** | 2 | NA | 2 | 1 | 1 | NA | NA | 2 | NA | NA | 2 | 1 | 11 |
| zhang | **2011** | 2 | NA | 2 | 1 | 1 | NA | NA | 1 | NA | NA | 2 | 1 | 10 |
| Pormohammad | **2022** | 2 | NA | 2 | 2 | 1 | NA | NA | 2 | NA | NA | 2 | 2 | 13 |
| choi | **2009** | 2 | NA | 2 | 1 | 1 | NA | NA | 2 | NA | NA | 2 | 1 | 11 |

NA= not applicable

Q1= Clearly stated aims/objectives

Q2= Detailed explanation of sample size calculation

Q3= Detailed explanation of sampling technique

Q4= Details of comparison group

Q5 = Detailed explanation of methodology

Q6= Operator details

Q7= Randomization

Q8= Method of measurement of outcome

Q9= Outcome assessor details

Q10= Blinding

Q11= Statistical analysis

Q12= Presentation of results
